# Simultaneous Infrared Spectroscopy, Raman Spectroscopy, and Luminescence Sensing: A Multispectroscopic Analytical Platform

**DOI:** 10.1021/acsmeasuresciau.1c00048

**Published:** 2021-12-27

**Authors:** Sarah Klingler, Julian Hniopek, Robert Stach, Michael Schmitt, Jürgen Popp, Boris Mizaikoff

**Affiliations:** † Institute of Analytical and Bioanalytical Chemistry, Ulm University, Albert-Einstein-Allee 11, Ulm, 89081, Germany; ‡ Department of Spectroscopy/Imaging, Leibniz-Institute of Photonic Technologies, Jena, 07745, Germany; § Institute of Physical Chemistry & Abbe Center of Photonics, Friedrich Schiller University Jena, Jena, 07743, Germany; ∥ Hahn-Schickard, Sedanstraße 14, Ulm, 89077, Germany

**Keywords:** infrared spectroscopy, attenuated total reflection, ATR, Raman spectroscopy, luminescence sensing, luminescence quenching, multispectroscopic platform, combined spectroscopic methods, H_2_O, D_2_O, ammonium sulfate, oxygen

## Abstract

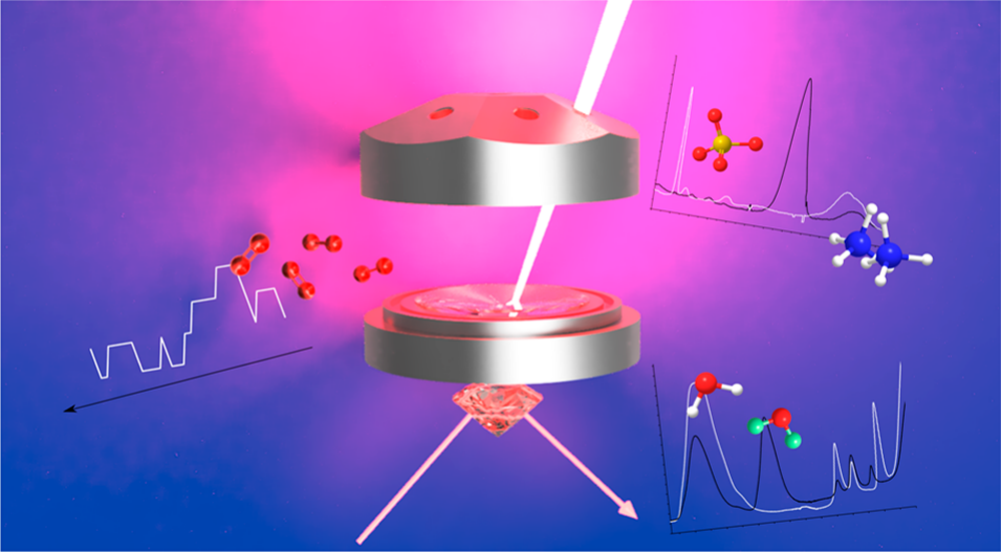

Scientific questions in fields such as catalysis, monitoring of biological processes, or environmental chemistry demand analytical technologies combining orthogonal spectroscopies. Combined spectroscopic concepts facilitate *in situ* online monitoring of dynamic processes providing a better understanding of the involved reaction pathways. In the present study, a low-liquid-volume multispectroscopic platform was developed based on infrared attenuated total reflection (IR-ATR) spectroscopy combined with Raman spectroscopy and luminescence sensing. To demonstrate the measurement capabilities, exemplary analyte systems including water/heavy water and aqueous solutions of ammonium sulfate were analyzed as proof-of-principle studies. It was successfully demonstrated that three optical techniques may be integrated into a single analytical platform without interference providing synchronized and complementary data sets by probing the same minute sample volume. In addition, the developed assembly provides a gastight lid sealing the headspace above the probed liquid for monitoring the concentration of molecular oxygen also in the gas phase via luminescence quenching. Hence, the entire assembly may be operated at inert conditions, as required, for example, during the analysis of photocatalytic processes.

## Introduction

For many state-of-the-art analytical questions including reaction pathway monitoring during photocatalysis or biological processes, rapid and time-resolved tracking of molecular structures, composition, and quantities is a prerequisite. Mid-infrared (MIR) spectroscopy has matured into one of the most prevalent analytical techniques for monitoring molecular processes owing to its inherent selectivity, nondestructiveness, and rapid data acquisition capabilities. Thus, molecular information on a wide range of organic and inorganic species may be obtained. Consequently, IR-ATR is frequently used as an analytical tool for the detection, identification, and quantification of molecules in the gas, liquid, and solid phase.^1,2^ Owing to the excitation of specific vibrational, ro-vibrational, and rotational modes, characteristic spectral patterns are obtained enabling qualitative and quantitative analysis, and providing access to chemical and structural characteristics, that is, “fingerprinting” any molecular species. The use of Fourier transform infrared (FTIR) spectrometers facilitates time-resolved studies for *in situ* and online monitoring, for example, in process control, during reaction pathway elucidation, and in environmental analysis.^1,3−5^

Attenuated total reflection spectroscopy is among the most flexible sampling techniques in this domain, especially for condensed phase applications with pronounced background matrix absorptions (e.g., water in aqueous solutions), as the evanescent field only probes a few micrometers into the sample above the internal reflection element (IRE) surface.^6−8^ While a variety of IRE materials are available, diamond is most beneficial taking advantage of its chemical inertness (e.g., against acids and bases), physical resilience, and mechanical robustness next to its advantageous refractive index providing a useful penetration depth of the evanescent field.^7,9^

During the present study, we have developed an analytical assembly combining the benefits of IR-ATR spectroscopy with its complementary vibrational spectroscopic counterpart, Raman spectroscopy. Next to IR spectroscopy, Raman spectroscopy has matured into a likewise commonly applied vibrational spectroscopic technique in chemical and biological analysis.^7,10,11^ As both techniques are based on different physical principles concerning the excitation of molecular vibrations (i.e., IR spectroscopy relies on a change of the molecular dipole moment, while Raman scattering requires a change in molecular polarizability), vibrations may be active or inactive in IR vs Raman or—alternatively—less or more pronounced in either technique. Thus, IR and Raman spectroscopy provide complementary molecular characteristics.^10−13^

Besides Raman and IR-ATR spectroscopy, a third optical technique was integrated into the multispectroscopic platform, that is, luminescence sensing. This adds the opportunity of precisely quantifying the oxygen concentrations in the gas and the liquid phase, which plays an important role in a wide variety of monitoring scenarios including industrial process monitoring, environmental monitoring (e.g., quality control of water^14,15^), and in photocatalysis (e.g., determination of the turnover number (TON) during water oxidation reactions^16^).^17−19^ As oxygen does not provide IR-active vibrations, and Raman spectroscopy is frequently not sensitive enough for gas phase oxygen sensing—and even less so for analyzing dissolved molecular oxygen—an additional optical technique was integrated, that is, oxygen sensing based on luminescence quenching effects. Dynamic quenching of the luminescence of appropriate dyes has matured into one of the most commonly applied techniques for the detection and quantification of molecular oxygen, in particular using fiberoptically coupled sensing techniques with tip-immobilized dyes. Next to an exceptionally fast response time, this detection principle does not consume oxygen, is inherently selective and robust, and enables measurements even in hard-to-reach places. Hence, luminescence-based oxygen sensors have several advantages versus conventional techniques.^12,17−22^

The particular development goal of the present study was establishing a methodology that facilitates improving fundamental understanding of photocatalytic reaction pathways along with the involved molecular changes of the photocatalyst and/or the embedding immobilization matrix. As each of the presented *in situ* spectroscopic techniques (i.e., IR, Raman, and luminescence quenching) provide individual advantages and disadvantages, the combination offers a powerful tool to overcome the drawbacks and to benefit from the strengths of each method.^23^ Raman spectroscopy provides advantages such as the comparative insensitivity toward water background signals.^24−28^ This complements IR-ATR spectroscopy that is much more affected by aqueous matrices.^11^ However, depending on the excitation wavelength Raman spectroscopy may suffer from a significant fluorescence background convoluting the vibrational signatures.^10^ Consequently, combining IR and Raman spectroscopy provides orthogonal information even facilitating potential *in situ* validation when simultaneously operated at the same sample.^24^

To clarify reaction mechanisms in, for example, photocatalytic reactions and to determine active species *in situ*, spectroscopic methods and especially their combination are relevant for future investigations at *in-operando* conditions. *In situ* spectroscopy enables deriving information on reaction intermediates and structural changes during the reaction with temporal resolution.^23,29,30^ Useful applications of combined *in situ* spectroscopic techniques (i.e., involving Raman, UV–vis, FBRM, IR-ATR, etc.) have been shown for a variety of scenarios providing supporting information on reaction pathways and in process control (e.g., crystallization processes,^25,28^ catalytic reactions,^24,23^ polymorphic transformations,^26,31−33^ etc.). “Gold standard” methods such as mass spectrometry, X-ray absorption spectroscopic methods (i.e., X-ray absorption near edge structure (XANES) or extended X-ray absorption fine structure (EXAFS)), or magnetic resonance spectroscopic methods (i.e., electron para-magnetic resonance (EPR), and NMR) were applied for studying several types of catalytic and (bio)chemical reactions in the liquid phase, yet are in part limited by extended measurement times and a lack of compactness and robustness for integration.^23,24,34^

Especially 2D data correlation methods benefit from the combination of orthogonal techniques. As a relatively young research field introduced by Noda in 1993, homo- and heterospectral 2D correlation techniques have evolved into effective and powerful tools for extracting spectroscopic information from complex data sets and minute molecular changes. By spreading the spectral information across two dimensions, systematic changes in data sets recorded under external perturbation may readily be obtained.^35^ Earlier research by our team and collaborators has successfully shown the clarification of catalytic reaction pathways via the heterogeneous correlation of Raman and IR-ATR spectra, however, not yet simultaneously recorded at the same sample.^36^ Hence, ensuring comparable reaction conditions (e.g., signal integration time, sample preparation, temperature, ambient conditions, etc.) results in extended experimental efforts. This further motivates the development of a combined platform integrating different spectroscopic techniques for ensuring the same analysis conditions facilitating data fusion and/or correlation.

In the present approach, a multispectroscopic measurement platform was developed based on a single-bounce diamond IRE ATR assembly coupled to a conventional FTIR spectrometer integrating fiberoptic Raman spectroscopy into the sampled volume. In addition, fiberoptically coupled luminescence quenching sensors for determining oxygen was integrated to monitor the integrity of the lid sealing, and the stability of headspace conditions. Background stability is essential, in particular if small changes—here, in oxygen concentration—should be observed, for example, during future photocatalytic conversions. To demonstrate the utility of the developed assembly, two exemplary yet relevant measurement scenarios were selected. Mixtures of H_2_O and D_2_O were evaluated, as deuteration is an important modulator for the elucidation of reaction pathways.^5,37^ Additionally, various concentrations of ammonium sulfate were analyzed owing to its vibrational spectrum with differently pronounced vibrational features in IR and Raman spectra along with its relevance in environmental sensing scenarios (e.g., as component in fertilizers).^38,39^

## Experimental Section

### Multispectroscopic Measurement Platform

**Figure 1:**
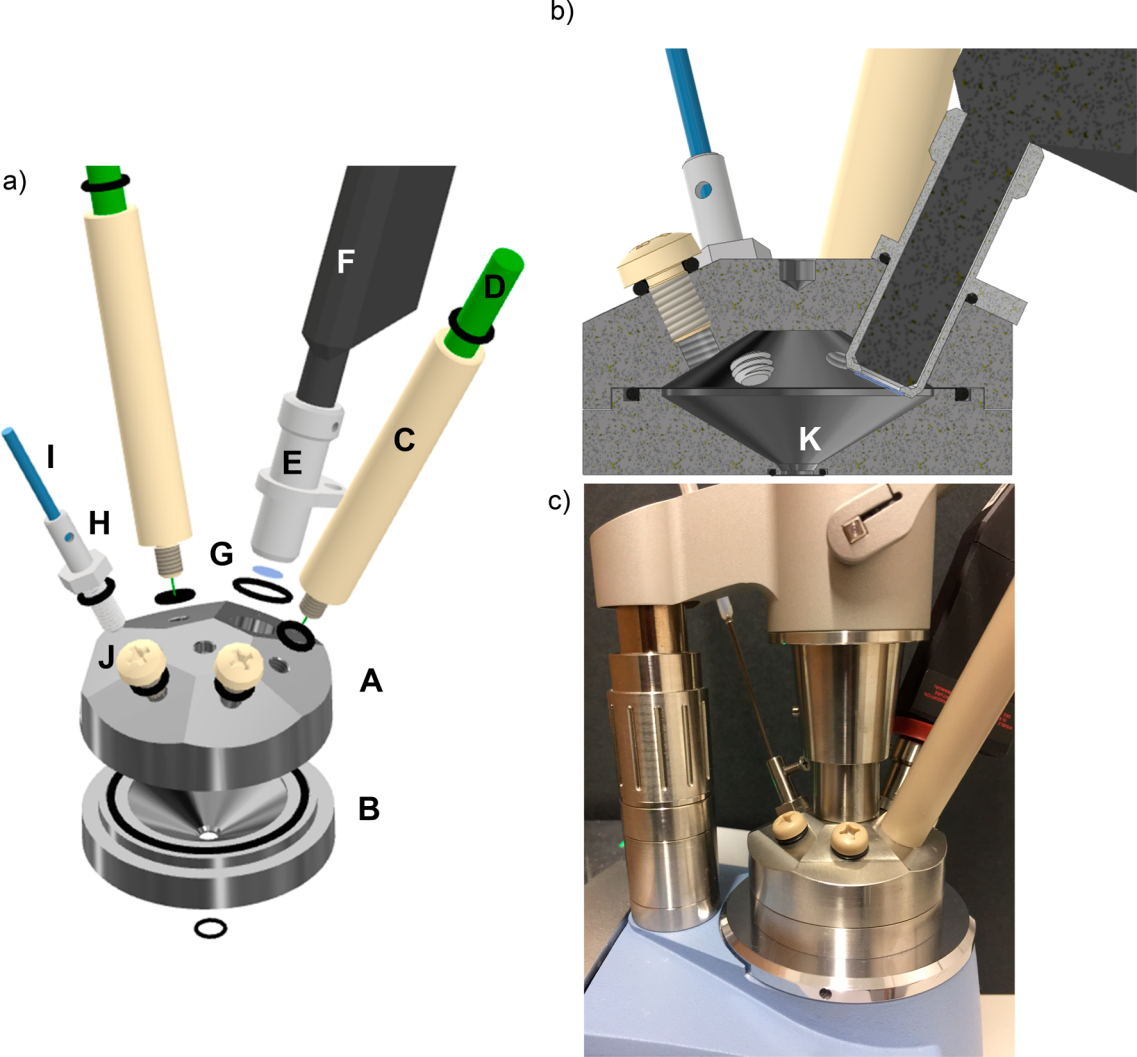
Schematic (CAD rendering) of the developed multispectroscopic measurement assembly. Exploded drawing (a), cross-sectional view of the sample chamber (b), and image of the assembly (c) attached to a compact FTIR spectrometer.

Figure 1a shows the developed multispectroscopic measurement platform. The system was fabricated from stainless steel (VA 1.4301) and consists of two parts: (i) a lid (A) with five tapped holes (M6) (J) for inserting different fiberoptically coupled sensors gastight via thread adapters, and (ii) a lower part (B) with a circular aperture with conical shape (4.5 mm diameter) at the bottom (K) (detail see Figure 1b) facilitating IR-ATR measurement via a single-bounce diamond IRE. An additional port (12 mm diameter) to house the Raman probe was included. For the insertion of the sensors, the lid provides six milled surfaces (at an angle of 25° each) providing a flat surface for sealing via O-rings and ensuring a suitable probe angle for the analysis. Stainless steel provides robustness against most chemicals (e.g., acids, etc.), oxidation stability and corrosion resistance, while also avoiding background fluorescence that may affect the Raman measurements. The fluidic cell requires a small sample volume of only 7 mL. All parts were sealed gastight using Viton (FPM75) O-rings (black rings). For inserting the Raman probe (F) (RIP-RPB-785-FC-SMA, Ocean Optics B. V, EW Duiven, Netherlands), a customized adapter (E) was developed and fabricated from stainless steel (VA 1.4301), and sealed via an optical window made from sapphire (G) (0.4 mm thickness × 7.5 mm diameter) glued with epoxy resin. As the Raman probe has a converging lens with a focal length of 7.5 mm, the adapter had to be inserted into the liquid for optimal sensor response. For inserting other sensors in a gastight fashion, customized adapters were developed and fabricated (C, H) as needed. For inserting the temperature sensor (I), again stainless steel VA 1.4301 (H) was used ensuring thermal conductivity and chemical stability. For integrating two fiberoptic oxygen sensors (D; one for the gas phase, one for the liquid phase) PEEK (C) was utilized. The temperature sensor (Pt100 temperature probe, TDIP15, PyroScience GmbH, Aachen, Germany) (I) was included, as oxygen measurements via luminescence quenching are strongly dependent on temperature.^19^ The multispectroscopic measurement platform was established adapting a Platinum Diamond ATR module (Bruker Optik GmbH, Ettlingen, Germany) connected to a compact FTIR spectrometer (Alpha I OEM, Bruker Optik GmbH, Ettlingen, Germany) shown in Figure 1c.

### Measurement Procedure

An IR background spectrum was recorded after 7 mL sample solution was filled into the measurement cell via a pipet. The cell was then sealed with a Viton O-ring and a screw. A Raman dark background spectrum was also collected before turning on the laser and simultaneously executing all three optical measurements.

For the calibration of the oxygen sensors, a 2-point customized calibration procedure was executed. For the gas phase detection and for setting the 0% O_2_ point, an N_2_ atmosphere (99.999% N_2_, class 5.0, MTI IndustrieGase AG, Neu-Ulm, Germany) was used; for the second point, synthetic air (20.5% O_2_, 79.5% N_2_, MTI IndustrieGase AG, Neu-Ulm, Germany) was utilized. For the liquid phase oxygen calibration, N_2_ was used to sparge deionized water for approximately 6 min. For setting the zero-calibration-point, the gas flow was turned off and a hold time was executed until no gas bubbles were evident. Afterward, the aqueous matrix was purged with synthetic air and the same procedure was repeated to set the second calibration point.

### Data Acquisition

For IR data acquisition, the software package OPUS 8.5 (Bruker Optik GmbH, Ettlingen, Germany) was utilized. The IR spectra were recorded via a room temperature deuterated l-alanine doped triglycine sulfate (DLaTGS) detector in the spectral range of 400 to 4000 cm^–1^ at a spectral resolution of 2 cm^–1^, using a Blackman-Harris three-term apodization and a zero-filling factor of 2 averaging 52 scans for each spectrum. For Raman data acquisition and processing, a Maya2000Pro spectrometer (grating type, 600; slit size, 25 μm) and the software OceanView 1.6.7 (both Ocean Optics B.V., EW Duiven, Netherlands) were used with an integration time of 4000 ms for each scan averaging six scans, which resulted in a total integration time of 24 s. The excitation wavelength was 785 nm at a laser power of 900 mW (Ocean Optics B.V., EW Duiven, Netherlands). A boxcar smoothing width of 5 was used averaging over adjacent detector elements (i.e., averaging each data point with 5 points to the left and the right). The signals of the two luminescence sensors (Oxygen Retractable Microsensors OXR50, PyroScience GmbH, Aachen, Germany) were recorded using the software Pyro Oxygen Logger 3.31.8 (PyroScience GmbH, Aachen, Germany) in continuous data acquisition mode. Every 3 s one data point was recorded with data smoothing of 3 in both the gas and liquid phase processed by the FireSting-PRO (FSPRO-4) Oxygen Meter (PyroScience GmbH, Aachen, Germany).

IR, Raman, and luminescence measurements were started simultaneously.

### Data Evaluation

Data evaluation was performed using the spectroscopy software package eFTIR (Operant LLC) and Origin 2021 (OriginLab). 2D correlation analysis was performed using GNU R and custom R packages, which have been described in detail elsewhere.^40,41^ As the reference spectrum, the mean spectrum of the data set was selected in all cases.

To evaluate different mixtures of H_2_O/D_2_O (100% H_2_O; 100% D_2_O; H_2_O/D_2_O 9:1, 3:1, 1:1, 1:3) each measurement for the particular ratio was repeated five times.

For ammonium sulfate studies, each measurement was repeated 10 times, and 6 different concentrations were analyzed (0 M, 0.05 M, 0.10 M, 0.25 M, 0.50 M, 0.75 M, 1.00 M).

For IR and Raman spectral data evaluation of sulfate (SO_4_^2−^ and ammonium (NH_4_^+^), respectively, as well as for H_2_O and D_2_O concentrations, an integration method was developed. The respective spectral regions are summarized in Table 1.^42−46^

**Table 1:** Parameters for IR and Raman Spectral Evaluation

method	band	integration/cm^–1^	baseline correction/cm^–1^
IR	^d^SO_4_^2–^	1148–1058	1167–1023
	NH_4_^+^	1484–1424	1523–1380
	D_2_O^a^	2767–2181	2808–2170
	H_2_O^a^	3534–3058	2752–3802
Raman	^c^SO_4_^2–^	999–956	1032–901
	NH_4_^+^		
	D_2_O^a^	2768–2242	2807–2169
	H_2_O^a^	3654–2930	3771–2884

To evaluate the performance of the developed measurement platform with regard to water and deuterium oxide mixtures, also the intensity ratios were determined by calculating the ratio of the mean value of the peak intensity for 100% H_2_O and D_2_O for IR and Raman.

The mean averaged values of the integrated peak areas for SO_4_^2–^ and NH_4_^+^ were then normalized with respect to the maximum sulfate integral for better comparison and plotted against the concentration to perform a linear regression.

The determination of the limit of detection (LOD) and limit of quantification (LOQ) for both systems was performed according to the IUPAC 3σ/10σ criteria.

### Chemicals

Ammonium sulfate ((NH_4_)_2_SO_4_, for biochemistry) was obtained from Merck (Darmstadt, Germany); deuterium oxide (D_2_O, 99.9%) in analytical quality was purchased from Deutero GmbH (Kastellaun, Germany). Both commercially available chemicals were used without further purification. For dilutions, deionized water was utilized.

## Results and Discussion

The multispectroscopic measurement platform was evaluated in terms of functionality and during proof-of-principle studies providing orthogonal data, respectively.

### Analysis of Deuterium Oxide and Water

**Figure 2:**
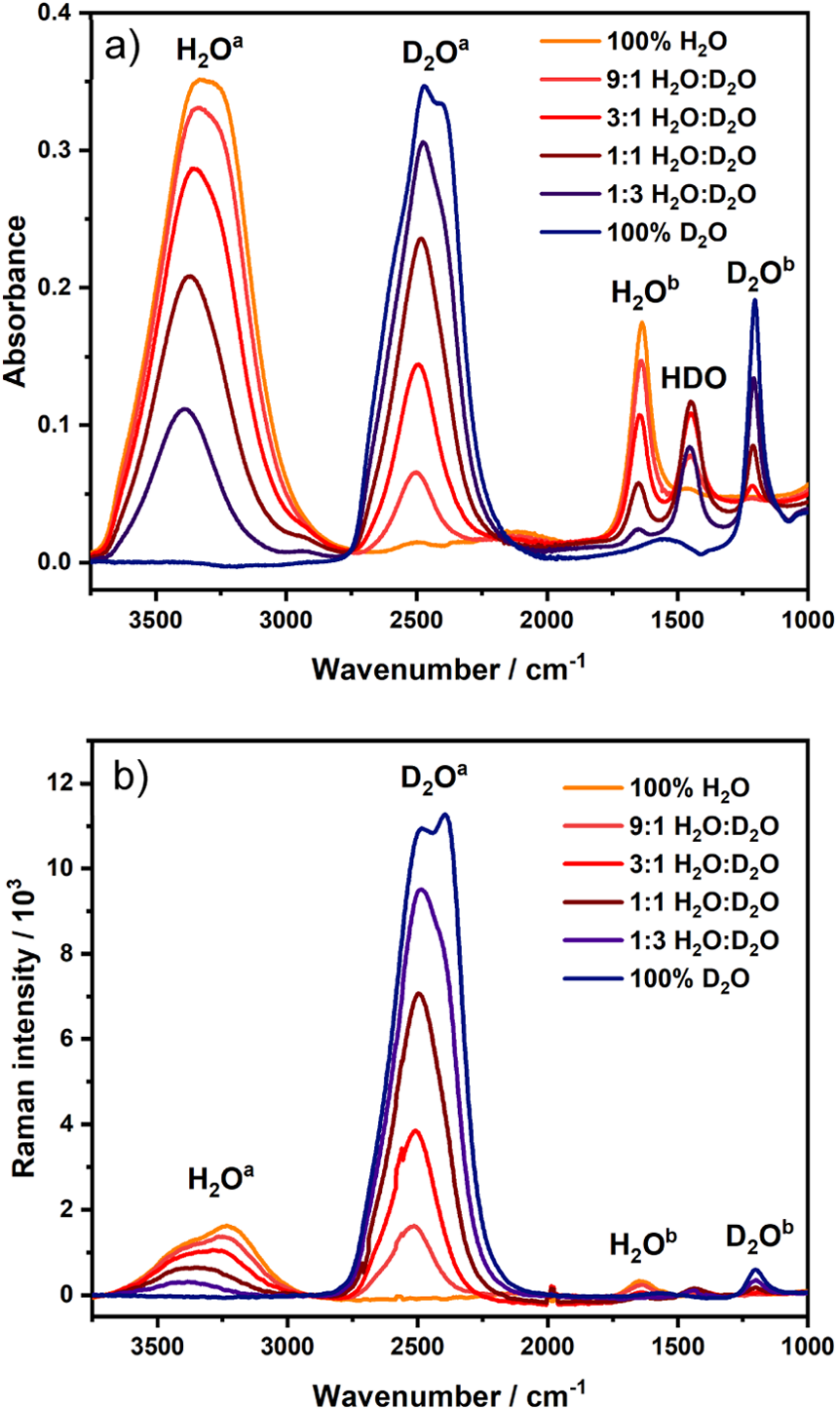
IR absorption spectra (a) and Raman spectra (b) of D_2_O/H_2_O mixtures. The stretching vibrations are marked with a superscripted “a”, the bending vibrations are labeled with “b”.

Deuteration is a common approach enabling nonradioactive isotope labeling. Thereby, complex reaction pathways or biological roots become traceable, especially also with vibrational spectroscopic techniques. To study the capability of the multispectroscopic platform for isotope tracking, different volume mixtures of D_2_O and H_2_O were analyzed. Figure 2 panels a and b show the absorption and Raman spectrum of different ratios. The rather broad bands can be explained by the coordination of water molecules in solution, that is, the effects of hydrogen bonding and intermolecular coupling (i.e., environmental broadening).^43,47^ The occurrence of the double peak for the symmetric stretching vibrations in the resulting IR and Raman spectra—for D_2_O^a^ at 2469 (IR) and 2484 cm^–1^ (Raman), respectively, and H_2_O^a^ 3323 (IR) and 3235 cm^–1^ (Raman)—at high D_2_O or H_2_O concentrations, respectively, is a result of the existence of non-hydrogen-bonded (NHB) and hydrogen-bonded (HB) molecules.^43^ As evident in the IR spectra, the intensity ratio of H_2_O in comparison to D_2_O equals 0.997, comprising similar IR sensitivities (i.e., change in dipole moment).^43,47^ In contrast, the intensity ratio H_2_O to D_2_O resulting for the Raman studies is 0.157 indicating that the Raman sensitivity to D_2_O is more than six-times higher than for H_2_O owing to the decrease in intrinsic scattering efficiency at higher wavenumbers (i.e., ω^4^ dependency) and the decreased detector sensitivity for exceedingly long wavelengths (3000 cm^–1^ = 1025 nm @ 785 nm excitation wavelengths). By evaluating the LOD and LOQ, this behavior is again evident, as summarized in Table 2. The LOD of D_2_O for Raman measurements is twice as high as for IR-ATR experiments, and almost eight-times higher than for H_2_O. This indicates that the Raman measurements are less affected by high water content within the sample matrix. Furthermore, the Grotthuss mechanism (i.e., the molecular pathway through which protons or deuterons are exchanged between water molecules) could indeed be tracked via the HDO signal.^37^

**Table 2:** Comparison of the LOD and LOQ for H_2_O and D_2_O for Raman and IR Measurements (*n* = 5)

method	band	LOD/%	LOQ/%
IR	D_2_O^a^	1.5	4.4
	H_2_O^a^	2.1	6.4
Raman	D_2_O^a^	0.7	2.2
	H_2_O^a^	5.5	16.7

Isotope labeling facilitates a better understanding of reaction pathways, which clearly benefits from a combined IR/Raman approach. The benefit of labeling via the exchange of deuterium and hydrogen atoms has been demonstrated in a variety of scenarios using IR-ATR spectroscopy, for example, for elucidating reaction pathways in environmental, biochemical, or catalytic reactions.^5,37^ Deuterium labeling appears even more effective for Raman measurements, as the D_2_O band is significantly more intense and pronounced in comparison to the OH stretching vibration in IR. In contrast, the OH stretching vibration is of the same intensity as the OD stretching vibration. Thus, the combined approach benefits from different sensitivities toward the vibrations caused by the isotopic labeling. In addition, these measurements also demonstrate that the simultaneous execution of the IR and Raman measurements do not result in any signal interference (i.e., the measurements do not influence each other). Last but not least, this combined approach also offers the opportunity of *in situ* validation of data with an independent technique, should this be of interest.

Bending vibrations—H_2_O^b^ and D_2_O^b^ at 1636 and 1208 cm^–1^ (IR), and at 1639 and 1204 cm^–1^ (Raman)—are more intense in the IR spectra vs Raman measurements. Here, the different sensitivity toward particular vibrational modes for the two spectroscopic methods is once again evident. Thus, the combined measurements indeed act as orthogonal methods offering the opportunity to extend the range substances and vibrational modes that may be analyzed.

### Analysis of Ammonium Sulfate

In a second example, the capabilities of combined Raman and IR measurements for validation and orthogonality were demonstrated. Therefore, a variety of concentrations of ammonium sulfate in deionized water (0, 0.05, 0.10, 0.25, 0.50, 0.75, and 1.00 M) were analyzed. Again, LOD and LOQ for each technique were derived.

**Figure 3:**
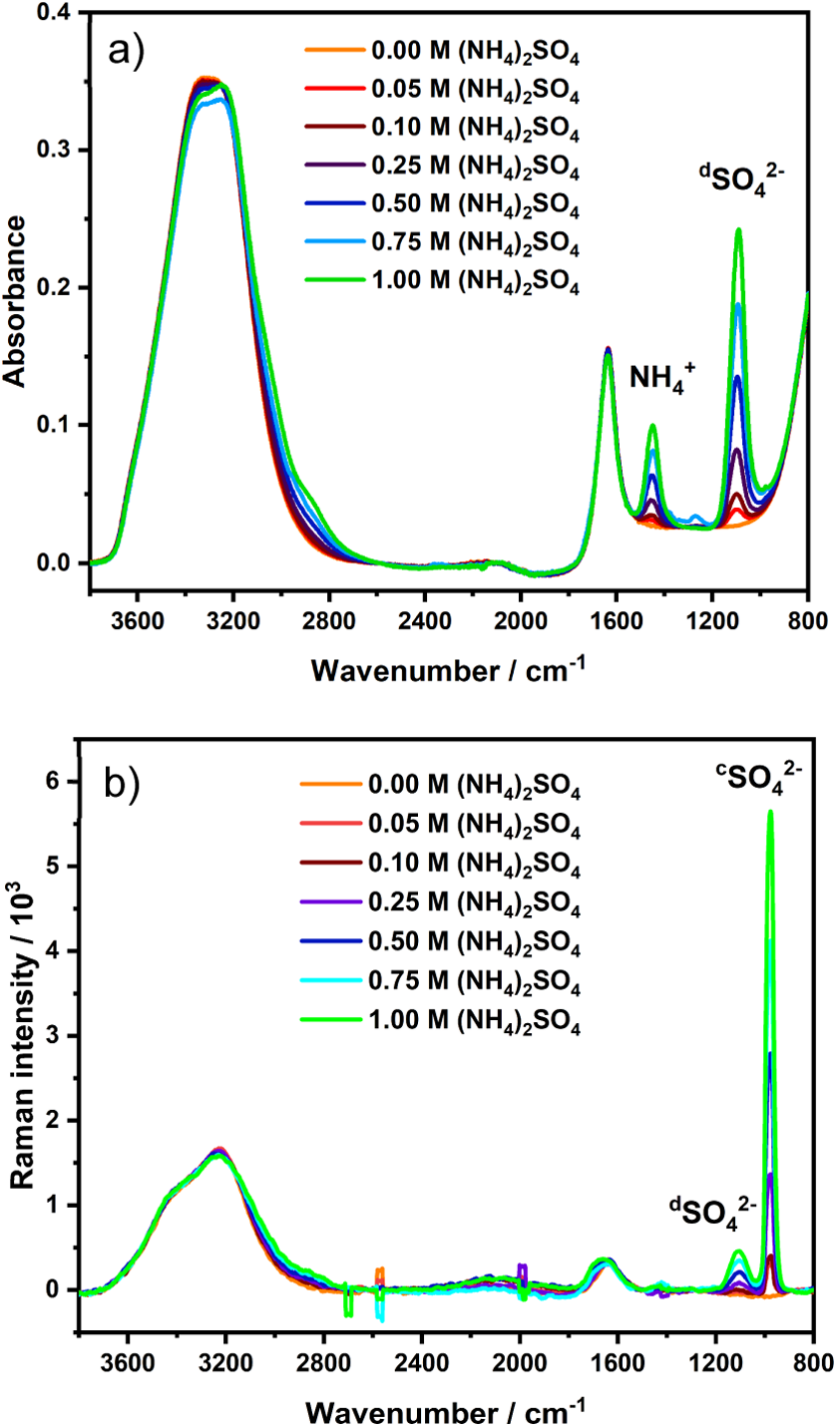
IR absorption spectra (a) and Raman spectra (b) of different concentrations of ammonium sulfate in deionized water. The vibrational modes of the sulfate ion are labeled with a superscripted “c” (ν_1_(*A*_1_)) and “d” (ν_3_(*T*_2_)), respectively.^38^

Figure 3 shows exemplary spectra for both IR (a) and Raman measurements (b) with the evaluated absorption bands (^c^SO_4_^2–^, ^d^SO_4_^2–^, and NH_4_^+^). Obviously, the IR spectra show a strong water background across the entire spectra, while the Raman spectra are less influenced by water depicting clearly separated water bands at 3228 cm^–1^ and 1646 cm^–1^, respectively. Thus, Raman measurements provide less signal overlap in the spectral regions where, for example, amide bands are occurring, which is beneficial/important for, for example, biochemical applications studying proteins.^48^ Furthermore, Figure 3b shows a second sulfate peak ^d^SO_4_^2–^ with less intensity near the analyzed sulfate band ^c^SO_4_^2–^. The “free” and undistorted SO_4_^2–^ ion has a tetrahedron symmetry (*T*_*d*_) associated with nine active intramolecular vibrations that are all Raman active. In contrast, only the *T* modes (i.e., ν_3_(*T*_2_) and ν_4_(*T*_2_)) are IR active.^38^ Thus, for the performed calibration several vibrational modes of the sulfate ion were evaluated (see Figure 3). As there are vibrational modes that are active in both IR and Raman or only in one of the two methods, respectively, and frequently exhibit differences in intensity, the developed multispectroscopic platform again benefits from increased access to different vibrational modes via IR and Raman, which may prove particularly important in complex matrices.

**Figure 4:**
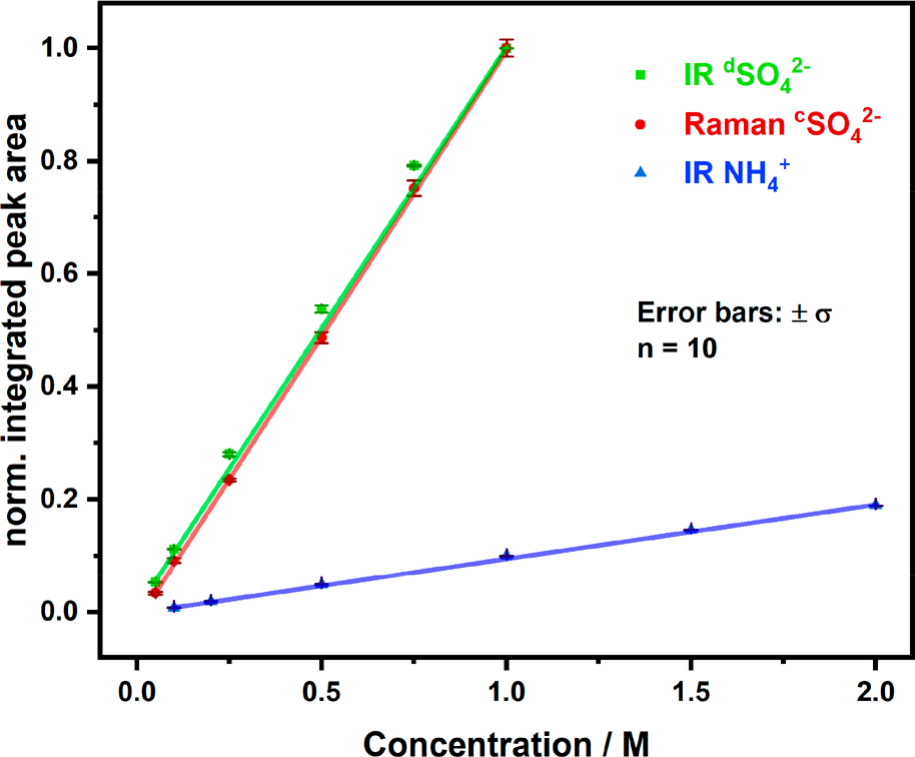
Calibration functions for sulfate and ammonium via simultaneous *in situ* IR and Raman spectroscopy.

As shown in Figure 4, the calibration function of SO_4_^2–^ shows agreement between the IR and the Raman measurements. Both slopes *m* of the linear regressions are almost equal, that is, *m*(SO_4_^2–^, Raman) = 1.01 ± 0.01 and *m*(SO_4_^2–^, IR) = 1.00 ± 0.01, respectively, indicating similar selectivity toward the sulfate ion although caused by different vibrational modes. The coefficient of determination is *R*^2^ = 0.9998 for both linear regressions confirming excellent quality of the calibration functions in the examined concentration range. Once more, it is highlighted that the orthogonal application of two methods not only enables the interference-free identification and quantification of different molecular vibrations and thus species, but also allows validating the results with two independent methods. As both methods may be applied *in situ* and at the same sample, also time may be saved for cross-checking data, which is an inherent benefit of the developed platform. Since both methods provide different spectral ranges where overlaps between the background matrix and the target analytes may occur, most spectral windows are available for evaluating target species without matrix interferences.

For IR, the LOD and LOQ was determined at 0.005 and 0.015 M, respectively, for sulfate. In contrast, the Raman measurements provide 6-fold higher values, 0.031 and 0.095 M. The LOD and LOQ for the ammonium ion with IR was 0.287 and 0.872 M, respectively. The sensitivity for detecting ammonium and its quantification via the two spectroscopic methods was significantly lower, which is evident by the lower slope *m*(NH_4_^+^, IR) = 0.096 ± 0.001 (*R*^2^ = 0.9996). For the Raman measurements, no ammonium peak could be evaluated. Here the two methods provide complementary data and differences in sensitivity. If a particular species is not detectable by Raman spectroscopy in the observed concentration range, IR spectroscopy may be used and vice versa. This flexibility adds to the versatility of the developed analytical platform, especially in complex molecular scenarios.

### Oxygen Monitoring

**Figure 5:**
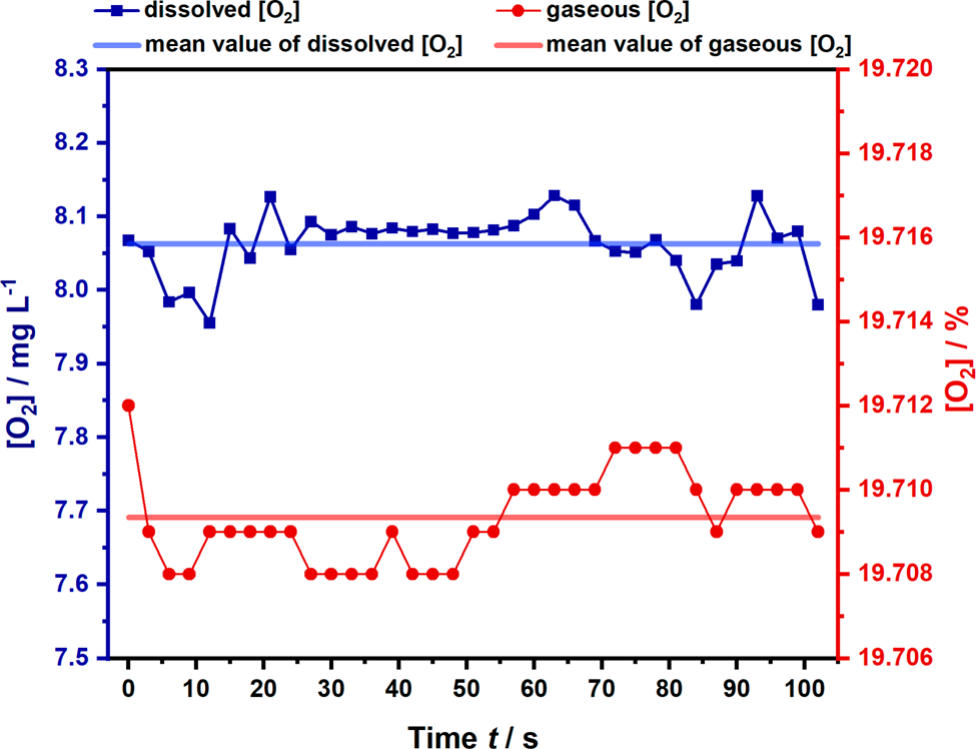
Luminescence sensor response of sensor 1 probing the headspace above the liquid sample (red), and sensor 2 immersed in the aqueous/liquid phase (blue).

Oxygen measurements were performed during each measurement. Figure 5 shows exemplarily the luminescence sensor response during a measurement period of 100 s. The red line indicates the oxygen concentration in the gas phase, while the blue line shows the oxygen concentration monitored in the liquid phase. The straight line represents the calculated mean value of the respective measurement series during 100 s. As the exemplary molecular systems analyzed herein do not cause any change of the oxygen concentration in both liquid and gas phase, the oxygen concentration should—and indeed does—remain constant during the measured time, that is, only slightly fluctuating around the mean value. This also indicates that the developed sampling cell is indeed gastight and providing excellent capabilities for future operation at inert conditions without influence of the ambient environment. It should be noted that monitoring of the gas phase is of particular importance as a complementary indicator for compositional changes in the liquid phase, for example, during photocatalytic conversion studies. For example, the *in situ* determination of the turnover number (TON) during water oxidation reactions^16,49^ or the monitoring of the oxygen concentration in water quality analysis^15^ relies on precise and continuous oxygen measurements, ideally without the need of expensive, off-line, and in part analyte- and time-consuming analytical techniques such as gas chromatography (GC), electrochemical probes (e.g., Clark electrode), pressure transducers or volumetric methods, which in addition require diligent sampling and sample preparation.^50^

### 2D Correlation Analysis

2D correlation analysis especially benefits from a combined measurement assembly for the simultaneous collection of heterospectral data sets. This significantly simplifies the process of ensuring equal measurement conditions for the spectral moieties, which is an essential prerequisite for the application of 2DCOS. To this end, we applied heterospectral 2D correlation analysis to the data sets recorded in this study to demonstrate the possible application of this technique in future more challenging scenarios.

**Figure 6:**
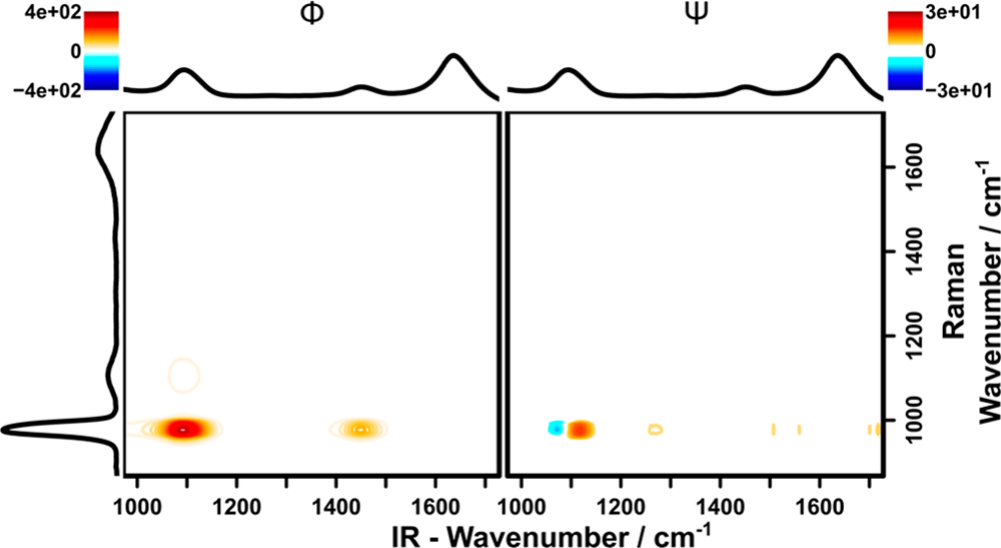
Synchronous (left) and asynchronous (right) hetero-2D correlation spectrum of ammonium sulfate solution between 0 and 1 M in water. The *x*-axis shows the IR spectrum, the *y*-axis shows the Raman spectrum.

Figure 6 depicts the hetero-2DCOS obtained by correlating the Raman- and the IR data obtained in this study. As expected, the synchronous correlation spectrum is dominated by the intense sulfate band in the Raman spectrum at approximately 980 cm^–1^, which is correlated with the sulfate and ammonium signals at 1100 and 1450 cm^–1^, respectively. This indicates a synchronous, that is, correlated change of these bands, which is different for, for example, the water band at 1640 cm^–1^. While this is of course immediately evident for a simple system such as pure ammonium sulfate, in a more complex sample these highly correlated peaks would offer the opportunity to identify chemically related species within the spectra, even if they are highly convoluted.

The asynchronous 2DCOS plot provides further insight into the multimodal measurement concept presented herein. Since 2DCOS is especially sensitive to minute differences in the perturbation dependency of signals, a simple one-component experiment as shown here may serve as an ideal benchmark to test for undesired, for example, instrument-induced dependencies, since for a noninteracting one component system, no asynchronicity is expected. The asynchronous 2DCOS plot (Figure 6, right) shows one significantly asynchronous signal around (1100, 990) cm^–1^, and additional smaller signals along the *y* = 990 cm^–1^ parallel. Since these smaller signals do not have a corresponding signal in the synchronous spectrum, they can be attributed to noise residues and may thus be ignored from a molecular perspective. The signal at (1100, 990) cm^–1^ can be explained by a shift of the asymmetric sulfate vibration signal to lower wavenumbers with increasing concentration, as reported in literature.^51^ Such a band shift exerts itself in a characteristic positive–negative pattern in the asynchronous 2DCOS signal (a.k.a., “butterfly” pattern). This is therefore a signal based on chemical signatures, and not induced, for example, by the instrument. The absence of additional asynchronicity demonstrates that the developed multispectroscopic assembly provides an excellent tool for recording multimodal data sets without introducing differences in signal dependency between the two modalities

**Figure 7:**
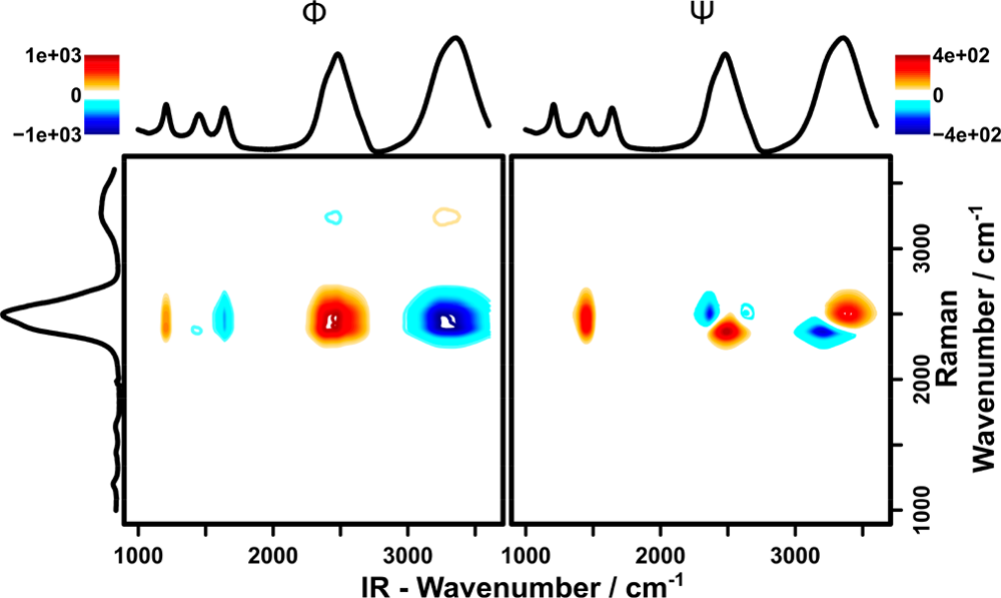
Synchronous (left) and asynchronous (right) hetero-2D correlation spectrum of mixtures between H_2_O and D_2_O. The *x*-axis shows the IR spectrum, the *y*-axis shows the Raman spectrum.

The H_2_O/D_2_O mixtures shows the potential of 2DCOS for discriminating uncorrelated and correlated changes between the complementary techniques, IR and Raman spectroscopies. Figure 7 depicts the synchronous and asynchronous hetero-2DCOS spectra of this data set. The synchronous 2DCOS is once again dominated by correlation patterns with the most intensive Raman band (D_2_O^a^) at 2484 cm^–1^ owing to its 9-fold higher intensities, as compared to the respective H_2_O^a^ band. In addition, less intense correlation patterns can also be found along this line around *y* = 3235 cm^–1^. Positive correlation patterns of the D_2_O band with the corresponding D_2_O-associated band in the IR spectrum (2469, 1209) cm^–1^, as well as negative correlation patterns with the H_2_O-associated bands at 3323 and 1653 cm^–1^ are evident. The inverse observation holds true for the correlation with the H_2_O^a^ band, although here, only the patterns correlated with the stronger stretching vibrations are clearly evident. Importantly, the correlation pattern with the HDO bending vibration, which would be expected at (1453, 2484) cm^–1^ is only weakly pronounced. This provides a first hint that the changes at these spectral positions, that is, the increase in D_2_O signal and increase in HDO signal are not strongly correlated, and therefore, cannot be attributed to the same species.

Combining the synchronous with the asynchronous spectrum (Figure 7, right) definitely confirms the assumptions derived from the synchronous spectrum. While for the correlation patterns of the bending vibrations (IR) of H_2_O and D_2_O with the D_2_O^a^ stretching vibration (Raman) no asynchronicity occurs, a strong asynchronous cross-peak stretches around (1453, 2484) cm^–1^, that is, the D_2_O/HDO correlation. This clearly shows that the changes at these spectral positions cannot be associated with the same species. In an unknown system, such information is indispensable to ensure correct assignment of bands and the associated changes in molecular species. In this multimodal setup, this could for example be used to associate IR signals that are highly convoluted with a strong and well-known Raman band of a target species, therefore enabling the tracking of changes to this species even in a convoluted sample matrix.

Furthermore, the asynchronous spectrum once again reveals two “butterfly” patterns associated with the D_2_O^a^ stretching vibration, and with the stretching vibrations of H_2_O and D_2_O in the IR spectrum. These patterns once again result from band shifts of the stretching vibrations due to the change in hydrogen-bonding environment depending on the D_2_O/H_2_O ratio. These effects are known from literature^37^ and therefore once again indicate molecular information rather than instrument-induced artifacts.

## Conclusions and Outlook

The present study demonstrates the successful integration of three optical measurement techniques into a single measurement assembly enabling simultaneous *in situ* IR-ATR, Raman, and luminescence quenching measurements in a sample volume of 7 mL providing complementary and/or validating information, respectively.

The sample cell was designed such that a modular adaption/addition of a variety of optical techniques to a basic assembly of a single-bounce diamond IR-ATR device coupled to an FTIR spectrometer is facilitated. The modularity of the concept enables that essentially any fiberoptically coupled Raman spectrometer may be used, as the cell lid and the probe adapters may be readily modified according to the dimensions of the utilized probe. The platform is also constructed in a way that other fiberoptically coupled tools such as light sources (e.g., LEDs with different emission wavelengths, etc.) could be readily inserted. This approach may be used for triggering, for example, light-driven reactions, as required for studying photocatalysis in molecular detail. The gas-tightness of the assembly provides the opportunity for facilitating inert reaction conditions (e.g., working under argon, etc.), as frequently required during catalytic reactions, but also for precisely determining the evolved oxygen concentration when determining TONs or when monitoring changes in oxygen levels as an indicator of compositional changes in solution.

Furthermore, the achieved analytical figures-of-merit evidence that the obtained measurements—because of the robustness of the developed multispectroscopic assembly—are highly reproducible, as confirmed by the quality of the obtained calibration functions and by the absence of instrument-attributed asynchronicity in the 2D correlation analysis results. Two exemplary molecular systems—H_2_O/D_2_O and dissolved ammonium sulfate—were selected to verify the utility and functionality of the system. In addition, the developed approach allows for *in situ* validations via complementary yet independent analytical techniques (i.e., IR vs Raman) without time-consuming additional experiments. The orthogonality of the achieved results is clearly evident for both exemplary systems, as there are different Raman and/or IR active bands also varying in intensity, respectively. Thus, different spectral overlaps of matrix and target analyte bands are provided, which expands the interference-free spectral range that may be used for analyzing complex molecular mixtures via the developed multispectroscopic platform.

During future studies, the developed platform will be used for online monitoring of environmental pollution scenarios, and for *in situ* following molecular changes during light-driven photocatalytic reactions. The modular system concept also allows operating the sample cell in a flow-through mode enabling dynamic studies at controlled flow conditions. Furthermore, it is anticipated that a similar device will be adapted for replacing conventional FTIR techniques with quantum/interband cascade laser-based spectroscopies combined with thin-film waveguide technology promising further enhanced sensitivity in even smaller sample volumes, as fundamentally shown during previous studies of our research team.^1,6,37^

Last but not least, the simultaneous execution of orthogonal spectroscopies at the same sample clearly simplifies data fusion approaches, as well as using (heterogeneous) 2D correlation methods (i.e., IR and Raman data) for extracting more information from complex data sets, as already demonstrated for the model systems herein. The simultaneous execution of the measurements ensures that the same experimental and/or reaction conditions in terms of temperature, light power, ambient conditions, etc. are present, which is a prerequisite for useful 2D correlation analysis, and can be applied for more realistic and complex systems in the future.
